# Resolvin D1 Suppresses H_2_O_2_-Induced Senescence in Fibroblasts by Inducing Autophagy through the miR-1299/ARG2/ARL1 Axis

**DOI:** 10.3390/antiox10121924

**Published:** 2021-11-30

**Authors:** Hyun Ji Kim, Boram Kim, Hyung Jung Byun, Lu Yu, Tuan Minh Nguyen, Thi Ha Nguyen, Phuong Anh Do, Eun Ji Kim, Kyung Ah Cheong, Kyung Sung Kim, Hiệu Huy Phùng, Mostafizur Rahman, Ji Yun Jang, Seung Bae Rho, Gyeoung Jin Kang, Mi Kyung Park, Ho Lee, Kyeong Lee, Jungsook Cho, Hyo Kyung Han, Sang Geon Kim, Ai Young Lee, Chang Hoon Lee

**Affiliations:** 1 BK21 FOUR Team and Integrated Research Institute for Drug Development, College of Pharmacy, Dongguk University, Seoul 04620, Korea; ev4444@dongguk.edu (H.J.K.); qtrami@gmail.com (B.K.); bhj1052@dongguk.edu (H.J.B.); yulu@dongguk.edu (L.Y.); nmtuan28@dongguk.edu (T.M.N.); nguyenthiha@dongguk.edu (T.H.N.); dophuonganh@dongguk.edu (P.A.D.); star0661@dongguk.edu (K.S.K.); 2020126666@dgu.ac.kr (H.H.P.); mostafizur@dgu.ac.kr (M.R.); yun9230@ncc.re.kr (J.Y.J.); mkpark@ncc.re.kr (M.K.P.); kaylee@dongguk.edu (K.L.); neuroph@dongguk.edu (J.C.); hkhan@dongguk.edu (H.K.H.); sgkim@dongguk.edu (S.G.K.); 2 Lillehei Heart Institute, University of Minnesota, Minneapolis, MN 55455, USA; kim00662@umn.edu; 3 Department of Dermatology, Dongguk University Ilsan Hospital, 814 Siksa-dong, Ilsandong-gu, Goyang-si 10326, Korea; bionase@hanmail.net (K.A.C.); kang0268@umn.edu (G.J.K.); lay5604@naver.com (A.Y.L.); 4 National Cancer Center, Goyang 10408, Korea; sbrho@ncc.re.kr (S.B.R.); ho25lee@ncc.re.kr (H.L.)

**Keywords:** ARG2, miR-1299, ARL1, autophagy, cell senescence, fibroblast, H_2_O_2_

## Abstract

ARG2 has been reported to inhibit autophagy in vascular endothelial cells and keratinocytes. However, studies of its mechanism of action, its role in skin fibroblasts, and the possibility of promoting autophagy and inhibiting cellular senescence through ARG2 inhibition are lacking. We induced cellular senescence in dermal fibroblasts by using H_2_O_2_. H_2_O_2_-induced fibroblast senescence was inhibited upon ARG2 knockdown and promoted upon ARG2 overexpression. The microRNA miR-1299 suppressed ARG2 expression, thereby inhibiting fibroblast senescence, and miR-1299 inhibitors promoted dermal fibroblast senescence by upregulating ARG2. Using yeast two-hybrid assay, we found that ARG2 binds to ARL1. ARL1 knockdown inhibited autophagy and ARL1 overexpression promoted it. Resolvin D1 (RvD1) suppressed ARG2 expression and cellular senescence. These data indicate that ARG2 stimulates dermal fibroblast cell senescence by inhibiting autophagy after interacting with ARL1. In addition, RvD1 appears to promote autophagy and inhibit dermal fibroblast senescence by inhibiting ARG2 expression. Taken together, the miR-1299/ARG2/ARL1 axis emerges as a novel mechanism of the ARG2-induced inhibition of autophagy. Furthermore, these results indicate that miR-1299 and pro-resolving lipids, including RvD1, are likely involved in inhibiting cellular senescence by inducing autophagy.

## 1. Introduction

Autophagy is a physiological process that regulates the breakdown and recycling of waste as well as of damaged intracellular components through lysosomal activity. The regulation of autophagy plays an essential role in tissue homeostasis and aging [1]. The suppression of autophagy is regarded as a phenotype of aging [2]. For example, autophagy-related proteins, such as Atg5, Atg7, and beclin1, are expressed at lower levels in the aged human brain than in the young brain [3]. In addition, urothrin-A–induced mitophagy and caloric-restriction-induced autophagy have been reported to prolong lifespan and maintain health [4,5,6].

Type II-arginine:ureahydrolase, arginase-II (ARG2), is a mitochondrial enzyme that is inducible and widely expressed in many extrahepatic tissues/cells [7,8,9]. The function of ARG2 is to hydrolyze L-arginine to urea and L-ornithine, thereby reducing the bioavailability of cellular L-arginine to vascular endothelial nitric oxide synthase (eNOS) and resulting in vascular dysfunction by producing vasoprotective nitric oxide [10,11]. L-ornithine acts as a biosynthetic precursor of the polyamines involved in cell growth and protein synthesis [12]. ARG2 is associated with nitric oxide synthase 3 (NOS3)/eNOS dysfunction in the pathogenesis of vascular aging and atherosclerosis [13]. Previous studies have shown that ARG2 stimulates the RPS6KB1 signaling independently of its enzymatic activity and thereby promotes endothelial senescence and inflammation [14] as well as the senescence and apoptosis of vascular smooth muscle cells [15]. ARG2 impairs endothelial autophagy through the modulation of the mTOR and PRKAA/AMPK signaling in advanced atherosclerosis [16]. Furthermore, several studies have shown that ARG2 is upregulated in senescent human and murine cells and can function independently of its enzymatic activity (i.e., a non-canonical effect) [14,15]. These studies have demonstrated that ARG2 induces mitochondrial dysfunction and apoptosis in vascular smooth muscle cells and that the mTORC1 and S6K1 signaling cascades provide evidence that mTOR activation impairs endothelial autophagy [15,16]. ARG2 also plays an important role in age-related vascular dysfunction [14,15,16].

The micro RNA miR-1299 is a tumor suppressor expressed in many cancer tissues [17,18,19]. Its expression level is lower in breast, ovarian, prostate, colon, cervical, liver, bile duct, and colon cancers than in the corresponding healthy tissues. This miRNA is a tumor suppressor and inhibits tumor-cell proliferation, invasion, and metastasis, improves chemotherapy sensitivity, and modulates tumorigenesis. Furthermore, the downstream target of miR-1299 is complex, indicating that it can regulate various target genes and, consequently, may have many roles in various diseases [20]. Recently, the miR-1299 target ARG2 was found to enhance the pigmentation in melasma by reducing melanosome degradation through the senescence-induced inhibition of autophagy [21].

This study aimed to investigate the inhibition of autophagy by ARG2, the target of miR-1299 in dermal fibroblast senescence, and the action of the pro-resolving RvD1 in the downregulation of ARG2 that results in cell senescence inhibition.

## 2. Materials and Methods

### 2.1. Materials and Plasmids

EMEM and fetal bovine serum (FBS) were obtained from the American Type Culture Collection (Manassa, VA, USA). DMEM, fetal bovine serum (FBS), phosphate-buffered saline (PBS), and penicillin/streptomycin; P/S were from Welgene Inc. (Gyeongsan-si, Korea). RvD1 was purchased from Cayman chemicals (Ann Arbor, MI, USA). The antibodies used were: β-actin (1:5000, sc-47778, Santa Cruz Biotechnology (SCB), Santa Cruz, CA, USA), ARL1 (1:1000, sc-393785, SCB), p53 (1:1000, sc-126, SCB), p21 (1:1000, #2947, Cell Signaling Technology (CST), Berkeley, CA, USA), p16 (1:1000, #18769, CST), ARG2 (1:1000, #19324, CST), Flag (1:1000, #14793, CST), HMGB-1 (1:1000, #6893, CST), p62 (1:1000, #23214, CST), LC3A/B (1:1000, #12741, CST). Secondary antibodies used were: anti-mouse HRP (1:5000, sc-2005, SCB), anti-rabbit-HRP (1:5000, SA002-500, GenDEPOT, Barker, TX, USA), anti-rabbit-Alexa488 (1:500, A21202, Thermo Fisher Scientific Inc., Waltham, MA, USA)), and anti-mouse-Alexa594 (1:500, A21203, Thermo Fisher Scientific Inc. Waltham, MA, USA). Hydrogen peroxide (#216763) and Acridine Orange (#235474) were purchased from Sigma-Aldrich (St. Louis, MO, USA).

### 2.2. Cell Culture

Human skin fibroblast cell line, BJ (CRL-2522, ATCC) was cultured in ATCC-formulated Eagle’s minimum essential medium (30-2003, ATCC) supplemented with 10% FBS and 100 U/mL penicillin, and 100 μg/mL streptomycin in T-75 flasks. Human primary dermal fibroblast was kindly gifted by Dr. Ai-Young Lee, Dongguk University. Cells were maintained in DMEM medium supplemented with 10% heat-inactivated FBS and 100 U/mL penicillin, and 100 μg/mL streptomycin. Cellmaxin (final concentration 5 μg/mL, C3314, GenDEPOT) was used to prevent mycoplasma contamination. The cells were grown at 37 °C in a humidified atmosphere containing 5% CO_2_.

### 2.3. siRNA or Plasmid DNA or microRNA Mimics and Inhibitors Transfection

For the gene-knockdown experiment, cells were transfected with the control or ARG2 small interfering RNA (siRNA; sc-29729, SCB) and ARL1 siRNA (sc-106957, SCB) using Lipofectamine™ 2000 Reagent (Invitrogen, Carlsbad, CA, USA) according to the manufacturer’s direction. The siRNA-to-Lipofectamine ratio was 1:1.2. For the transient overexpression study, cells were transfected with control or ARG2 (OHu20129D, Genescript, Piscataway, NJ, USA) or ARL1 (OHu02804, Genescript) plasmid vector. Approximately 70–80% confluent cells were transfected with Lipofectamine™ 2000 Reagent (Invitrogen) according to the manufacturer’s direction. Approximately 24–48 h after transfection, cells were used in further experiments.

Cell were transfected with control microRNA mimics (Dharmacon Research, Lafayette, CO, USA), human miR-1299 mimics (Dharmacon), negative control hairpin inhibitor, or miR-1299 hairpin inhibitor. The TransIT-siQUEST transfection reagent (Mirus, PanVera, Madison, WI, USA) was used for transfection according to the manufacturer’s protocol.

### 2.4. Cell Viability

Cells (1 × 10^4^ cells/well) were seeded into 96-well plates and were treated according to their indicated condition. After the indicated time, the supernatant was abandoned and 100 μL of CCK-8 (5 mg/mL, dojindo, Japan) dissolved in medium was added to each well and incubated for 1 h. The amounts of WST-formazan generated were measured on a microplate reader by absorbance at 450 nm using a Microplate Reader (BIO-RAD, Hercules, CA, USA).

### 2.5. β-Galactosidase Staining

Cells were seeded onto 6-well plates. Senescence-associated β-galactosidase staining was performed using a commercial kit (Senescence β-Galactosidase Staining Kit, #9860, CST), according to the manufacturer’s instructions. Staining was examined with a Leica microscope (Leica Microsystems, Wetzlar, Germany).

### 2.6. Acridine Orange Staining

The evaluation of autophagy by acridine orange staining was performed as previously described [22]. Briefly, cells were stained with 1 ug/mL acridine orange (Sigma-Aldrich) in PBS containing 5% FBS at 37 °C for 15 min. Cells were washed and then examined with a Leica fluorescence microscope (Leica Microsystems, Wetzlar, Germany).

### 2.7. Western Blot

Cells were washed twice with ice-cold PBS and disrupted in RIPA buffer containing Xpert Protease Inhibitor Cocktail Solution and Xpert Phosphatase Inhibitor Cocktail Solution (GenDEPOT) on ice for 10 min. Cell lysates were centrifuged at 15,000 rpm for 15 min at 4 °C, and supernatants subjected to Western blotting [23]. Total protein was quantified using the Pierce BCA Protein Assay Kit (Thermo Fisher Scientific Inc.). Proteins were separated by electrophoresis on an 8~10% sodium dodecyl sulfate-polyacrylamide gel (SDS-PAGE), after which samples were transferred onto polyvinylidene difluoride (PVDF) membrane. The membrane was treated with 5% skimmed milk for 1 h and incubated overnight at 4 °C with primary antibodies. After TBST washing, the membrane was incubated with the HRP-conjugated secondary antibody (1:5000) for 60 min at room temperature. The bands were visualized using West-Q ECL Solution (GenDEPOT).

### 2.8. Co-Immunoprecipitation

Cell lysate (1 mg) was incubated for 1 h with IgG and 10 μg of protein A/G beads [24]. The lysate was then incubated overnight at 4 °C with 10 μg of anti-ARG2 (CST.) and anti-ARL1 (SCB) antibodies or nonspecific IgGs. Following this, the lysate was incubated once more for 1 h at RT with 20 μg Pierce^TM^ protein A/G magnetic beads (Thermo Fisher Scientific Inc.). The samples containing the beads were placed in a magnetic separation rack and then the supernatants carefully removed. Pellets were washed with 1 mL of the lysate buffer (10 Mm Tris-HCl, pH7.4, 150 mM NaCl, 5 Mm EDTA, 0.1% Triton X-100). The immunoprecipitated proteins were extracted by 10 min boiling with 2x SDS-PAGE reducing sample buffer 40 μL and detected by immunoblot with anti-HIF1A or anti-Slug antibodies

### 2.9. Confocal Microscopy

Cells were seeded and incubated on coverslips. After transfection, the cells were washed with ice-cold PBS and fixed with 4% paraformaldehyde for 10 min at room temperature, prior to permeabilizing with 0.1% Triton X-100 for 10 min, blocking with 3% BSA for 30 min, and incubating with primary antibodies overnight at 4 °C. After PBS washing, cells were incubated with fluorescence-conjugated secondary antibodies for 1 h at RT. Finally, the samples were mounted onto slides with an UltraCruz^®^ aqueous mounting medium with DAPI (sc-24941, SCB) and visualized using confocal microscope system (Nanoscope, Daejeon, Korea).

### 2.10. Yeast Two-Hybrid (Y2H) Analysis

For bait construction with ARG2, full-length ARG2 was introduced into the EcoRI and XhoI restriction enzyme sites of the pGilda/LexA yeast shuttle plasmid vector. The resulting plasmid pGilda/LexA-ARG2 was transformed into yeast strain EGY48 (MATa, his3, trp1, ura3-52, leu2::pLeu2-LexAop6/pSH18-34 (LexAop-lacZ reporter)) [23]. The yeast cells containing pGilda/LexA- ARG2 were selected for the histidine prototrophy (pGilda/LexA plasmid marker) on synthetic medium (Ura^−^, His^−^) containing 2% (*w*/*v*) glucose. The full-length ARL1 was cloned into the pJG4-5 vector to generate B42 fusion proteins at EcoRI and XhoI sites. The ARL1 encoding pJG4-5/B42 fusion proteins were introduced into the competent cells that already contained pGilda/LexA- ARG2 and the transformants were selected for the tryptophan prototrophy (pJG4-5/B42 plasmid marker) on synthetic medium (Ura^−^, His^−^, Trp^−^) containing 2% (*w*/*v*) glucose. The binding activity of the positive interactions between the two-hybrid proteins were detected by cell growth on the leucine-depleted synthetic yeast medium containing 2% (*w*/*v*) galactose and by the formation of blue colony on the same synthetic medium containing 5 mM X-Gal (5-bromo-4-chloro-3-indolyl β-D-galactoside), 2% (*w/v*) galactose, and 2% (*w/v*) raffinose.

### 2.11. mCherry-GFP-LC3 Adenoviral Infection

An adenoviral construct encoding for mCherry-GFP-LC3 was kindly provided by Dr. Sang Geon Kim (Dongguk University) [25]. The cells were infected with mCherry-GFP-LC3-expressing adenovirus in DMEM containing 10% FBS for 24 h. For adenovirus infections, cells were deprived of serum for 4 h before harvest.

### 2.12. Luciferase Reporter Assay

pMIR-ARG2 and pMIR-mutant promoter was kindly gifted by Dr. Ai-Young Lee, Dongguk University. Cells were co-transfected with pMIR-ARG2 or pMIR-mutant and renilla vector with Lipofectamine™ 2000 Transfection Reagent (Invitrogen, Carlsbad, CA, USA) [21]. After the transfection, cells were rinsed in PBS. Cells were lysed in 250 μL of Passive Lysis 5X Buffer (PLB) provided in the kit, and the lysed solutions were incubated again for 15 min at room temperature with rocking. After the centrifugation at 12,000× *g* for 15 s at 4 °C, 20 μL of the supernatant was transferred to the white 96-well plate (SPL, Korea). A luciferase reporter assay was then performed using a Luciferase Assay System (Promega). The luciferase activity, expressed as relative light units, was normalized to renilla activity. The luciferase activities were measured using GloMax luminometer (Promega).

### 2.13. Statistical Analysis

The data are expressed as the mean ± S.E.M. of at least three independent experiments performed in triplicate. *p* < 0.05 was considered statistically significant.

## 3. Results

### 3.1. H_2_O_2_ Induces Cell Senescence in Skin Fibroblast Cells

**Figure 1:**
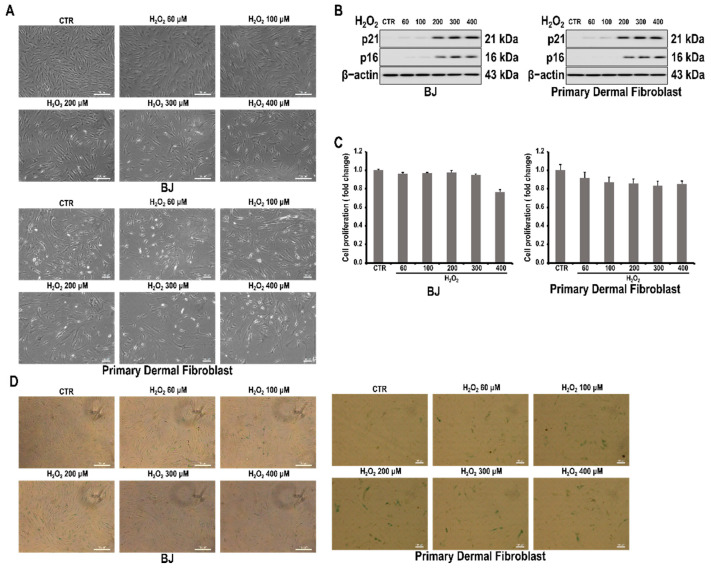
H_2_O_2_-induced cell senescence in skin fibroblast cells. Cells were treated with varying concentrations of H_2_O_2_ for 24 h. (**A**) Representative bright field images of skin fibroblasts after H_2_O_2_ treatment. (**B**) Effect of H_2_O_2_ on the expressions of p16 and p21 in skin fibroblasts. (**C**) Effect of H_2_O_2_ on the proliferation of skin fibroblasts, as determined by the MTT assay. (**D**) Representative images of H_2_O_2_-treated skin fibroblasts stained for senescence-associated β-galactosidase after H_2_O_2_ treatment.

When H_2_O_2_ was used to treat BJ skin fibroblasts at various concentrations, cells with senescent morphology, such as enlarged, flattened, and irregularly shaped cells, were observed (Figure 1A). Western blot results confirmed that the expressions of p16 and p21, which are cell senescence markers, also increased with H_2_O_2_ treatment (Figure 1B) [26]. The inhibitory effect of H_2_O_2_ on the proliferation was not significant up to 300 μM, and a decrease of 20% was observed at 400 μM (Figure 1C). Therefore, an H_2_O_2_ concentration of 200 μM was used for the subsequent cell senescence experiments. In addition, it was confirmed by β-gal staining that the activity of acidic β-galactosidase, known as a marker of cell senescence, was increased by treatment with H_2_O_2_ (Figure 1D).

### 3.2. ARG2 Is Involved in H_2_O_2_-Induced Cell Senescence in BJ Skin Fibroblasts

**Figure 2:**
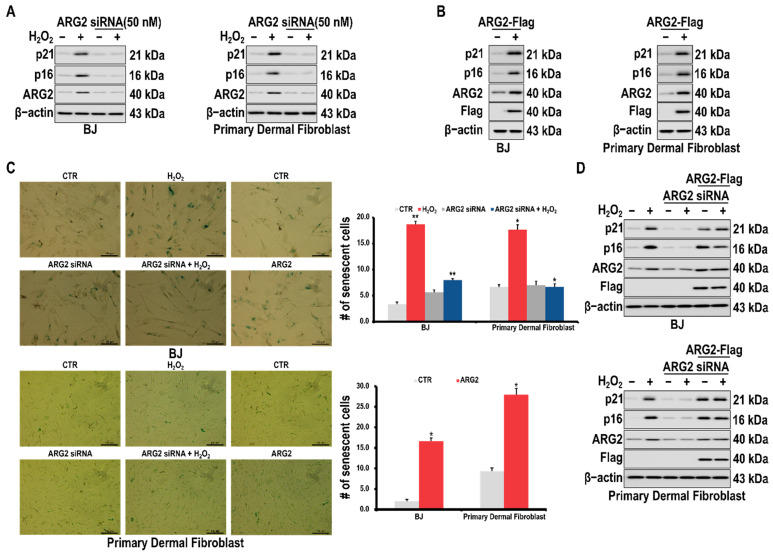
Effects of ARG2 on senescence of skin fibroblast cells. (**A**) Effect of ARG2 knockdown on the expressions of cellular senescence markers, p16 and p21, in skin fibroblasts. Cells were transfected with an ARG2 siRNA and then treated with H_2_O_2_ (200 μM) for 24 h. (**B**) Effect of ARG2 overexpression on the expressions of the cellular senescence markers, p16 and p21, in skin fibroblasts. Cells were transfected with a plasmid containing a flag-tagged ARG2. (**C**) Representative images of H_2_O_2_-treated skin fibroblasts stained for senescence-associated β-galactosidase after being transfected with an ARG2 siRNA (left) or an ARG2 overexpression plasmid (right). # represents the number of senescent cells. (**D**) Effect of restoring ARG2 levels on senescence of skin fibroblasts. Cells were transfected with an ARG2 siRNA, treated with H_2_O_2_ for 24 h, and then transfected with a flag-tagged ARG2 plasmid. Protein levels were determined using Western blotting. A * *p* value < 0.05 and ** *p* value < 0.01 were considered statistically significant and error bars represent ± SD.

To confirm whether ARG2 is involved in fibroblast senescence, BJ skin fibroblasts and primary dermal fibroblasts were treated with an ARG2 siRNA, and it was confirmed that the expression of the cellular senescence markers p16 and p21 was suppressed in the treated cells (Figure 2A). Conversely, p16 and p21 were upregulated when the ARG2 gene was overexpressed (Figure 2B). When ARG2 was knocked down by treatment with the ARG2 siRNA, the β-galactosidase activity was reduced (Figure 2C). Furthermore, the forced expression of ARG2 rescued the expression of p16 and p21 in the cells treated with the ARG2 siRNA (Figure 2D).

### 3.3. miR-1299 Is Involved in Cell Senescence by Regulating ARG2 Expression in Dermal Cells

**Figure 3:**
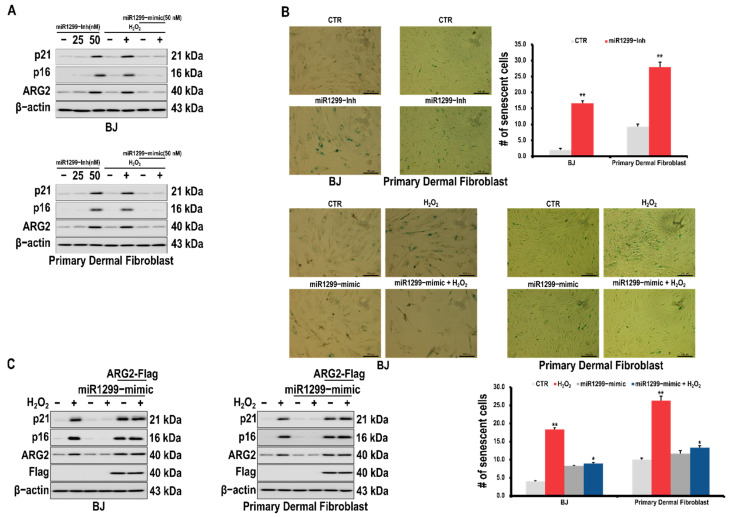
Effects of miR-1299 on the senescence of skin fibroblast cells. (**A**) Effect of the miR-1299 inhibitor and miR-1299 mimic on the expressions of cellular senescence markers, p16 and p21, in skin fibroblasts. Cells were transfected with an miR-1299 inhibitor and treated with H_2_O_2_ (200 μM) for 24 h after transfection. (**B**) Representative images indicating senescence-associated β-galactosidase in cells transfected with the miR-1299 inhibitor (left) or H_2_O_2_–induced skin fibroblasts transfected with an miR-1299 mimic (right). (**C**) Effect of restoring ARG2 expression on the senescence of skin fibroblasts transfected with miR-1299. Cells were transfected with an miR-1299 mimic and treated with H_2_O_2_ for 24 h after transfection and subsequently transfected with an ARG2 plasmid again. The protein levels were investigated using Western blotting. # represents the number of senescent cells. A * *p* value < 0.05 and ** *p* value < 0.01 were considered statistically significant and error bars represent ± SD.

miR-1299 is a microRNA that has been reported to inhibit the ARG2 expression [21]. ARG2, p16, and p21 were found to be upregulated after the cells were treated with an miR-1299 inhibitor and downregulated upon treatment with an miR-1299 mimic (Figure 3A). The expressions of p21 and p16 were found to be decreased upon H_2_O_2_-induced cellular senescence after treatment with the miR-1299 mimic (Figure 3A). Moreover, the activity of acidic β-galactosidase was observed to be increased after an miR-1299 inhibitor was administered to BJ fibroblasts; however, it decreased when the miR-1299 mimic was administered (Figure 3B). Reduction in cellular senescence caused by the addition of the miR-1299 mimic was found to be restored upon the overexpression of ARG2 (Figure 3C).

### 3.4. Interaction of ARG2 with ARL1 Suppresses Autophagy Leading to Cell Senescence

**Figure 4:**
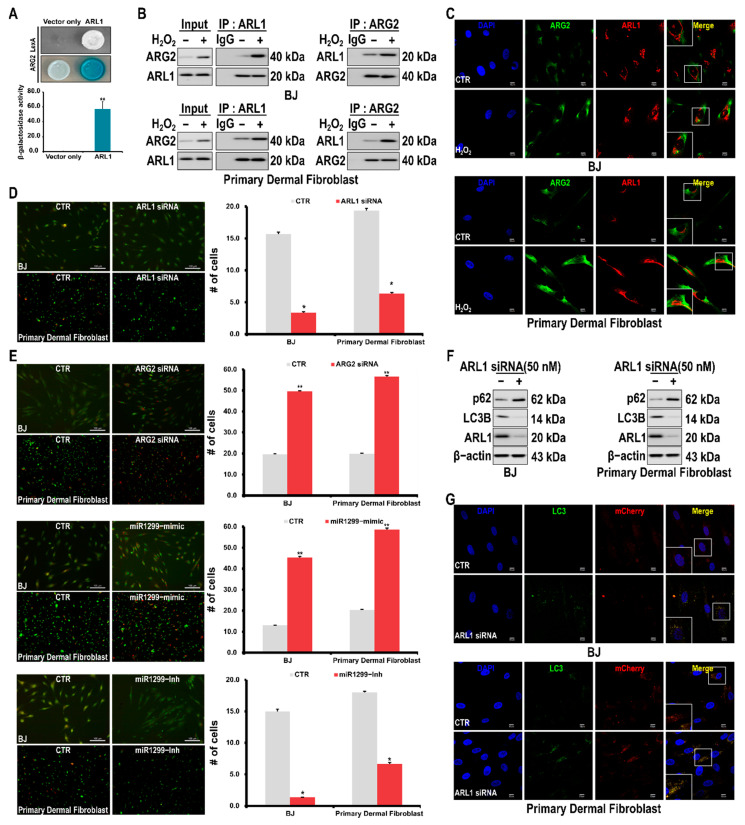
ARG2-induced senescence of skin fibroblasts through interaction with ARL1. (**A**) Yeast two-hybrid assay for ARG2 interaction with ARL1. (**B**) Co-immunoprecipitation of ARG2 with ARL1. Cells were treated with H_2_O_2_ (200 μM) for 24 h, and the interaction between ARG2 and ARL1 was examined by Western blotting. (**C**) Confocal microscopic analysis of ARG2 and ARL1 expression. Cells were treated with H_2_O_2_ for 24 h and examined by confocal microscopy. DAPI staining was used to identify the nucleus. Bar, 10 mm. (**D**) Effects of ARL1 knockdown on autophagy in skin fibroblasts. Cells were stained with acridine orange and examined by fluorescence microscopy. (**E**) Effects of ARG2 knockdown (top), miR-1299 mimic (middle), and miR-1299 inhibitor (bottom) on autophagy in skin fibroblasts. Cells were stained with acridine orange and examined by fluorescence microscopy. # represents the number of senescent cells. (**F**) Effect of ARL1 knockdown on the expressions of the autophagy-related proteins, p62 and LC3B, in skin fibroblasts. Protein levels were determined using Western blotting. (**G**) Effect of ARL1 knockdown on the autophagic flux in skin fibroblasts. Cells were transfected with an ARL1 siRNA and then transfected the next day with an adenoviral vector harboring tandem fluorescent mCherry-GFP-LC3 on the next day. After 24 h, the cells were examined by confocal microscopy. DAPI staining was used to identify the nucleus. Bar, 10 mm. A * *p* value < 0.05 and ** *p* value < 0.01 were considered statistically significant and error bars represent ± SD.

The mechanism by which ARG2 induces cellular senescence was investigated by examining the protein partners of ARG2 using the yeast two-hybrid assay. Consequently, ARL1 was found to bind to ARG2 (Figure 4A), which was confirmed by their co-immunoprecipitation in BJ and primary dermal fibroblasts (Figure 4B). The binding of these two molecules was also confirmed via confocal microscopy (Figure 4C).

ARG2 induces senescence by inhibiting autophagy [21]. ARL1 is involved in autophagy in yeast cells [26]. Therefore, we hypothesized that cellular senescence is promoted by the inhibition of autophagy since the binding of ARG2 to ARL1 interferes with the role of ARL1 in autophagy. Therefore, to determine whether the binding of ARL1 to ARG2 is involved in autophagy, ARL1 was knocked down, and the extent of autophagy was assessed via acridine-orange staining. Knocking down ARL1 reduced the number of cells undergoing autophagy (Figure 4D). As expected, the miR-1299 mimic increased the extent of autophagy that was observed after ARG2 knockdown, and the miR-1299 inhibitor decreased the number of cells undergoing autophagy (Figure 4E). The autophagy marker, LC3B, was found to be downregulated, whereas p62 was upregulated upon knocking down ARL1 (Figure 4F). Furthermore, decreased autophagy upon ARL1 knockdown was confirmed by using the mCherry-LC3-GFP reporter plasmid (Figure 4G). In contrast, the ARG2 siRNA and the miR-1299 mimic enhanced the autophagy (Supplement Figure S1).

### 3.5. RvD1 Inhibits H_2_O_2_-Induced Cellular Senescence in Fibroblast Cells through the miR-1299/ARG2/ARL1 Axis

**Figure 5:**
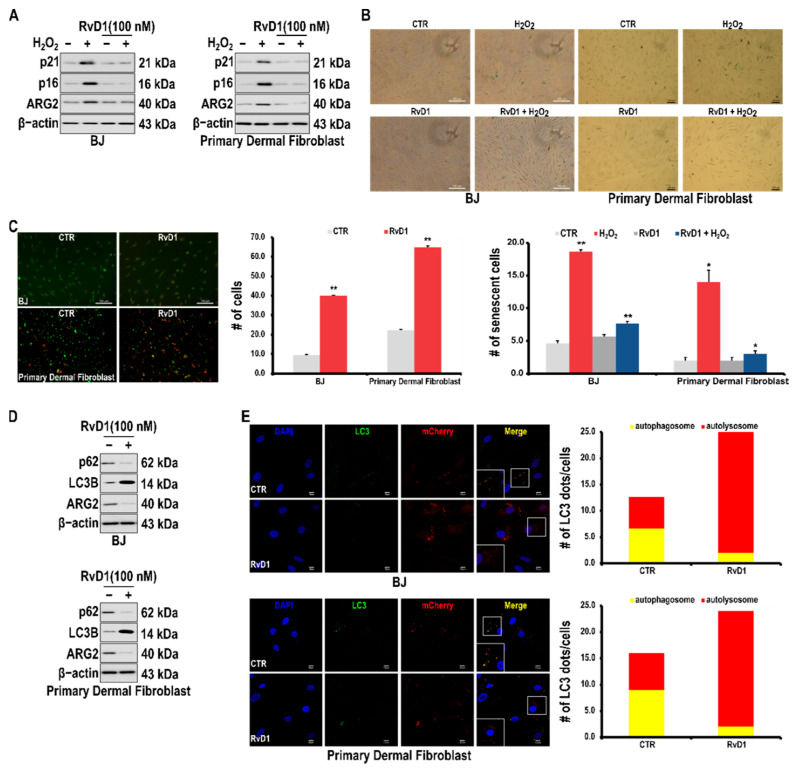
Effects of RvD1 on H_2_O_2_–induced cell senescence in skin fibroblasts. Cells were pretreated with RvD1 for 2 h, and subsequently treated with H_2_O_2_ (200 μM) for 24 h. (**A**) Effects of RvD1 on the expression of cellular senescence markers, p16 and p21, in skin fibroblasts. (**B**) Representative images of H_2_O_2_-induced skin fibroblasts treated with RvD1 stained for senescence-associated β-galactosidase. (**C**) Effect of RvD1 on autophagy in skin fibroblasts. Cells were stained with acridine orange and examined by fluorescence microscopy. (**D**) Effects of RvD1 on the levels of autophagy-related proteins, p62 and LC3B, in skin fibroblasts. Protein levels were determined using Western blotting. (**E**) Effect of RvD1 on the autophagic flux in skin fibroblasts. Cells were treated with RvD1 for 24 h, transfected for 24 h with an adenoviral vector harboring tandem fluorescent mCherry-GFP-LC3, and then analyzed using confocal microscopy. DAPI staining was used to identify the nucleus. Bar, 10 mm. A # represents the number of senescent cells. A * *p* value < 0.05 and ** *p* value < 0.01 were considered statistically significant and error bars represent ± SD.

We explored the possibility of inhibiting cellular senescence by factors regulating the expression of ARG2. We found that the pro-resolving lipid RvD1 downregulated ARG2 leading to the downregulation of p21 and p16 (Figure 5A). In addition, treatment with RvD1 inhibited the H_2_O_2_-induced increased activity of acidic β-galactosidase (Figure 5B). Accordingly, acridine orange staining showed that the number of cells undergoing autophagy increased upon RvD1 treatment (Figure 5C). In the cells treated with RvD1, as expected, p62 was downregulated and LC3B was upregulated (Figure 5D). Furthermore, increased autophagy upon RvD1 treatment was confirmed by using the mCherry-LC3-GFP reporter plasmid (Figure 5E). These results suggest the possibility that RvD1 inhibits cellular senescence by inducing autophagy via ARL1 through downregulation of ARG2.

## 4. Discussion

Cellular senescence refers to the stable cessation of cell proliferation. This process results from stresses, including oxidative stress, telomere attrition, and activated oncogenes [27]. The senescence-associated proliferative arrest is characterized by the activation of the TP53-CDKN1A/p21WAF1 and CDKN2A/p16INK4a-RB pathways. Senescent cells also increase lysosomal mass to increase senescence-associated GLB1/beta-galactosidase (SA-GLB1/β-gal) activity [28]. These cells are alive and primarily contribute to tissue stress responses through age-related secretory phenotypes [29,30]. The presented study reports whether the miR-1299 target ARG2 is involved in fibroblast senescence through its autophagy inhibitory action, its mechanism of action, and the effect of RvD1 as an inhibitor of ARG2 expression. For studying fibroblast senescence, BJ skin fibroblasts were treated with H_2_O_2_ to confirm their potential as a cellular senescence model. Changes in cell shape, the upregulation of p53 and p21, and the increased activity of acidic β-galactosidase were observed (Figure 1) [31]. These results are consistent with the results of other researchers and were used for subsequent experiments [32,33].

Kim et al. have reported that the upregulation of ARG2 in keratinocytes upon the downregulation of miR-1299 induces a decrease in melanosome degradation through decreased autophagy induced by senescence, thereby increasing pigmentation in melasma [21]. From these results, we questioned whether changes in the ARG2 level is related to cellular senescence in dermal cells and investigated the ARG2 expression during skin fibroblast senescence. We observed that ARG2 was upregulated in the H_2_O_2_-treated fibroblasts. In addition, the H_2_O_2_-induced upregulation of cellular senescence-related markers was inhibited by ARG2 knockdown and increased by ARG2 overexpression (Figure 2).

As expected, the downregulation in cellular senescence markers by ARG2 knockdown was suppressed by ARG2 overexpression (Figure 2D). These findings indicate that ARG2 is important in the observed fibroblasts, and that the enhanced activity of acidic β-galactosidase was also observed. (Figure 1) [31]. These results are consistent with the results of other researchers and were used for subsequent experiments [32,33].

Kim et al. reported that the upregulation of ARG2, in parallel to the downregulation of miR-1299 in keratinocytes decreases melanosome degradation by suppressing the senescence-induced autophagy, thereby increasing the pigmentation in melasma [21]. From these results, we questioned whether the fluctuation in ARG2 expression in dermal cells is related to cellular senescence. We observed that ARG2 was upregulated in the H_2_O_2_-induced fibroblast senescence model. In addition, the H_2_O_2_-induced upregulation of cellular senescence-related markers was inhibited upon ARG2 knockdown and increased upon ARG2 overexpression (Figure 2). As expected, the downregulation of these cellular senescence markers by ARG2 knockdown was reversed upon the forced expression of ARG2 (Figure 2D). These findings indicate that ARG2 is important in fibroblast senescence, consistently with the report that ARG2 is important in vascular aging. These findings further extend the importance of the role of ARG2 in cellular senescence [15,34].

Given that miR-1299 downregulates ARG2, we assessed whether this miRNA can inhibit H_2_O_2_-induced fibroblast senescence. The treatment of skin dermal cells with miR-1299 induced the downregulation of ARG2 and senescence-related markers. Furthermore, treatment with the miR-1299 inhibitor upregulated ARG2 and cellular senescence-related markers (Figure 3). There are no direct study results of the role of miR-1299 in cellular senescence except for the report by Kim et al. [19]. However, microRNAs are known to be major regulators of age-related diseases, and their involvement in longevity is emerging as a promising research topic [35,36]. Therefore, miR-1299 may serve as a promising anti-aging agent through the inhibition of ARG2 expression, and many follow-up studies are likely needed in the future.

Autophagy is inhibited by cellular senescence, and conversely, the proper induction of autophagy elicits antiaging effects. For example, autophagy determines the features of bone marrow mesenchymal stem cells and cellular senescence during bone aging [37]. Rapamycin, an autophagy inducer, can prevent the cellular senescence induced by p21 expression in humans without affecting the cell cycle [38]. Therefore, to determine how ARG2 inhibits autophagy, we performed yeast two-hybrid and co-immunoprecipitation assays and found that ARL1 binds to ARG2 (Figure 4A,B).

ARL1 has all the typical features of the Arf-family GTPases, including an amphiphilic N-terminal helix and a consensus site for N-myristoylation. In addition, both human and *Saccharomyces cerevisiae* ARL1 are substrates of N-myristoyltransferase [39]. ARL1 is believed to be a key protein in Golgi function in various eukaryotes [40,41,42,43]. This protein is present in the trans-membrane of the Golgi apparatus and can directly bind to and recruit the GRIP domain coiled-coil protein and recruit or activate Imh1, TGN46, and other effectors [42,44,45,46]. In yeast, Arl1 and Arl3 GTPases cooperate with Cog8 to regulate selective autophagy through Atg9 trafficking [22]. However, there have been no related studies on the possible involvement of ARL1 in autophagy in human cells. Therefore, our finding of ARL1 knockdown inhibiting autophagy in BJ fibroblasts shows for the first time that ARL1 is involved in autophagy in human cells (Figure 4).

ARG2-induced cellular senescence has been reported to not be related to the enzymatic activity of ARG2 [16]. Therefore, we tried to find a blocker of ARG2 expression instead of an ARG2 inhibitor. In this way, we expected that autophagy would be promoted and cellular senescence would be suppressed. Therefore, we investigated whether pro-resolving lipids that promote tissue homeostasis while inducing autophagy can inhibit the expression of ARG2 [47,48,49,50]. As expected, the pro-resolving lipid RvD1 suppressed the expression of ARG2 and H_2_O_2_-induced cellular senescence markers (Figure 5). As expected, RvD1 promoted autophagy in skin fibroblasts. The autophagy-promoting effect of RvD1 has also been reported in other cells such as macrophages and monocytes [49,51,52]. Therefore, our results suggest that RvD1 induces autophagy though a novel mechanism involving the miR-1299/ARG2/ARL1 Axis.

## 5. Conclusions

ARG2 induces cellular senescence in skin fibroblasts and this induction of cellular senescence is due to the inhibition of autophagy through binding with ARL1, which is involved in autophagy. In addition, RvD1, a pro-resolving lipid, seems to promote autophagy and suppress fibroblast senescence by downregulating ARG2. These results suggest that ARG2 and pro-resolving lipids such as RvD1 can be harnessed against senescence and potentially against aging.

## Data Availability

Data are contained within the article or supplementary material.

## Supplementary Materials

The following are available online at https://www.mdpi.com/article/10.3390/antiox10121924/s1, Figure S1: Effects of ARG2 knockdown and miR-1299 mimic on autophagic flux in skin fibroblasts
