# Supplementary figures and images for: Resolvin D1 Suppresses H2O2-Induced Senescence in Fibroblasts by Inducing Autophagy through the miR-1299/ARG2/ARL1 Axis
Source: Antioxidants (Basel). 2021 Nov 30;10(12):1924. doi: 10.3390/antiox10121924 (PMC8750589; doi:10.3390/antiox10121924)

Supplementary Figure 1

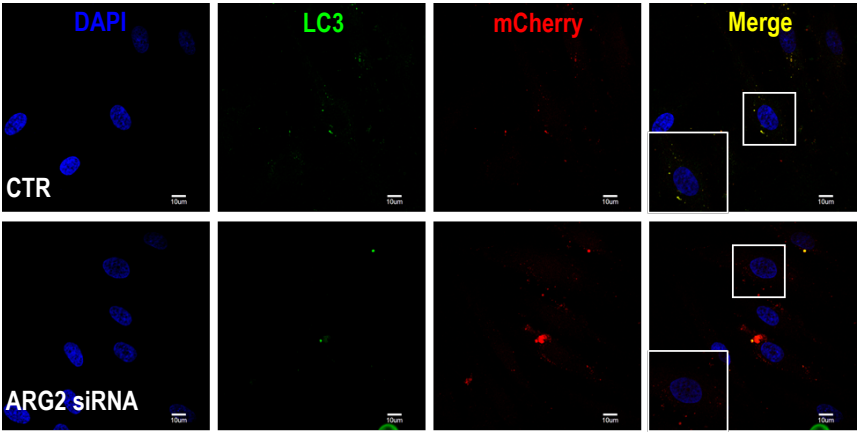

BJ

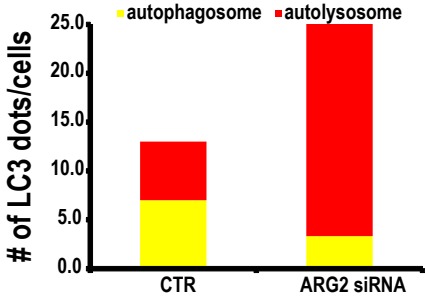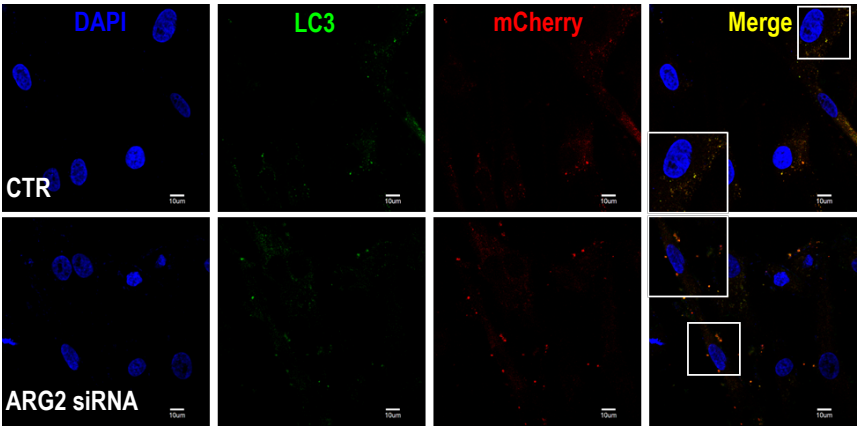

Primary Dermal Fibroblast

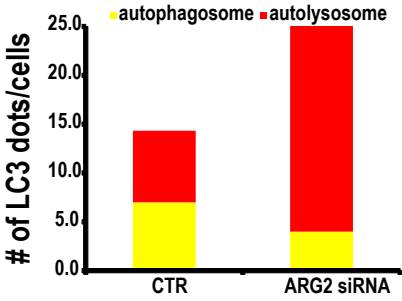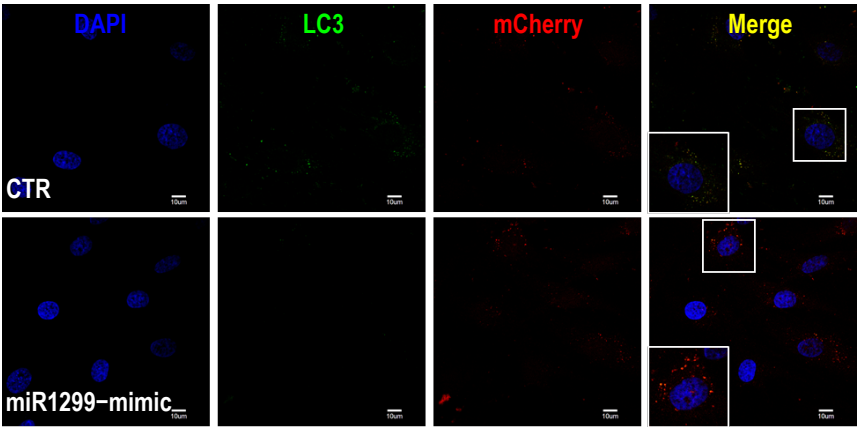

BJ

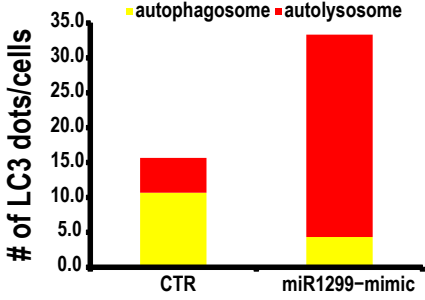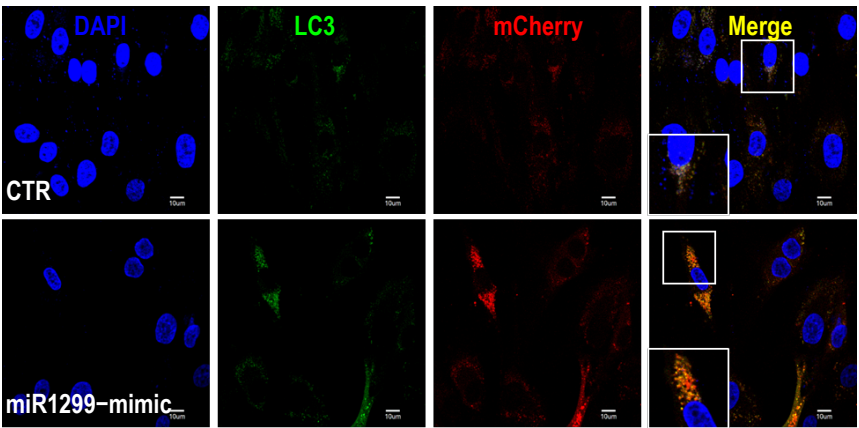

Primary Dermal Fibroblast

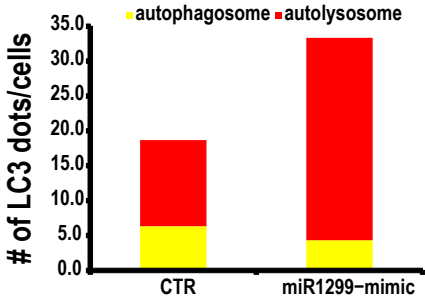

Supplement: Supplementary file 1 [file antioxidants-10-01924-s001.zip › antioxidants-1464366-supplementary.pdf]
